# Case Study: Aphonia in Psychosis: Bridging ENT and Psychiatry

**DOI:** 10.1192/bjo.2025.10755

**Published:** 2025-06-20

**Authors:** Akansha Tewari, Andrea Pathak, Ashish Pathak, Abhinav Tewari

**Affiliations:** 1 Dr. Ram Manohar Lohia Institute of Medical Sciences (RMLIMS), Lucknow, India; 2 Essex Partnership University NHS Foundation Trust (EPUT), Basildon, United Kingdom

## Abstract

**Aims:** Aphonia, defined as partial or complete loss of voice, can have multifactorial origin, ranging from organic to functional aetiology. This paper aims to describe an unusual presentation of aphonia on the background of psychotic symptoms and catatonia.

**Methods:** Mr KC, a 23-year-old mechanic presented to an outpatient ENT (Otorhinolaryngology) clinic at a tertiary care hospital in North India. As per available information, KC was asymptomatic until 1 month ago. Over the next week, the family members observed KC to be withdrawn, quieter than usual and skipped work. He would reluctantly accept food and eat less than usual. Family members consulted general practitioners who advised multivitamins and supportive measures, with no improvements in KC’s symptoms.

In the week preceding hospital visit, family members got concerned as KC would spend an entire day without speaking. He would occasionally nod yes/no or utter 1–2 words at his own will. He clenched his jaws when family attempted to feed him. Family googled KC’s symptoms and guided by the suggestions over the internet, they decided to visit the ENT clinic for further evaluation.

Upon presentation to the clinic, KC appeared dishevelled and had clearly lost weight, with his clothes hanging loosely. He refused cooperation for the oral and laryngoscopic examination. Neurological examination revealed increased rigidity in both limbs following which a psychiatry referral was done and a provisional diagnosis of catatonia was established.

After a lorazepam challenge, the rigidity reduced, and KC became more compliant, accepting small amounts of food. The laryngoscopy was performed when KC was cooperative, revealing no structural abnormalities or any pathological finding. His speech returned, and he expressed fear, saying, “I can hear people plotting against me”. With a diagnosis of psychosis, oral risperidone 4 mg per day (divided dose) was started. Routine blood tests revealed nutritional deficiencies likely due to prolonged food refusal. Two weeks into the treatment, KC resumed normal interaction, and his speech was fully restored.

**Results:** The case highlights the collaborative approach of ENT and mental health professionals in prompt identification and treatment of aphonia. Unlike many patients who present to ENT clinics with aphonia due to stress-related causes, this case is unique because psychotic symptoms overlapped with the aphonic presentation.

**Conclusion:** This case emphasizes the importance of a multidisciplinary approach in the diagnosis and management of aphonia. It serves as a reminder to consider a broader differential diagnosis when encountering aphonia, particularly in patients with unusual behavioural changes or neurological signs.

